# The temporal dependencies between social, emotional and physical health factors in young people receiving mental healthcare: a dynamic Bayesian network analysis

**DOI:** 10.1017/S2045796023000616

**Published:** 2023-09-08

**Authors:** Frank Iorfino, Mathew Varidel, Roman Marchant, Sally Cripps, Jacob Crouse, Ante Prodan, Rafael Oliveria, Joanne S. Carpenter, Daniel F. Hermens, Adam Guastella, Elizabeth Scott, Jai Shah, Kathleen Merikangas, Jan Scott, Ian B. Hickie

**Affiliations:** 1 Brain and Mind Centre, The University of Sydney, Sydney, NSW, Australia; 2 Human Technology Institute, University of Technology, Sydney, NSW, Australia; 3 School of Mathematical and Physical Sciences, University of Technology Sydney, Sydney, NSW, Australia; 4 Translational Health Research Institute, Western Sydney University, Sydney, NSW, Australia; 5 School of Computer, Data and Mathematical Sciences, Western Sydney University, Sydney, NSW, Australia; 6 School of Computer Science, The University of Sydney, Sydney, NSW, Australia; 7 Thompson Institute, University of the Sunshine Coast, Birtinya, QLD, Australia; 8 Department of Psychiatry, McGill University, Montreal, QC, Canada; 9 Genetic Epidemiology Research Branch, Division of Intramural Research Program, National Institute of Mental Health, Bethesda, MD, USA; 10 Academic Psychiatry, Institute of Neuroscience, Newcastle University, Newcastle, UK

**Keywords:** anxiety, causality, depression, functioning, mood disorders, personalised care, suicide ideation, youth

## Abstract

**Aims:**

The needs of young people attending mental healthcare can be complex and often span multiple domains (e.g., social, emotional and physical health factors). These factors often complicate treatment approaches and contribute to poorer outcomes in youth mental health. We aimed to identify how these factors interact over time by modelling the temporal dependencies between these transdiagnostic social, emotional and physical health factors among young people presenting for youth mental healthcare.

**Methods:**

Dynamic Bayesian networks were used to examine the relationship between mental health factors across multiple domains (social and occupational function, self-harm and suicidality, alcohol and substance use, physical health and psychiatric syndromes) in a longitudinal cohort of 2663 young people accessing youth mental health services. Two networks were developed: (1) ‘initial network’, that shows the conditional dependencies between factors at first presentation, and a (2) ‘transition network’, how factors are dependent longitudinally.

**Results:**

The ‘initial network’ identified that childhood disorders tend to precede adolescent depression which itself was associated with three distinct pathways or illness trajectories; (1) anxiety disorder; (2) bipolar disorder, manic-like experiences, circadian disturbances and psychosis-like experiences; (3) self-harm and suicidality to alcohol and substance use or functioning. The ‘transition network’ identified that over time social and occupational function had the largest effect on self-harm and suicidality, with direct effects on ideation (relative risk [RR], 1.79; CI, 1.59–1.99) and self-harm (RR, 1.32; CI, 1.22–1.41), and an indirect effect on attempts (RR, 2.10; CI, 1.69–2.50). Suicide ideation had a direct effect on future suicide attempts (RR, 4.37; CI, 3.28–5.43) and self-harm (RR, 2.78; CI, 2.55–3.01). Alcohol and substance use, physical health and psychiatric syndromes (e.g., depression and anxiety, at-risk mental states) were independent domains whereby all direct effects remained within each domain over time.

**Conclusions:**

This study identified probable temporal dependencies between domains, which has causal interpretations, and therefore can provide insight into their differential role over the course of illness. This work identified social, emotional and physical health factors that may be important early intervention and prevention targets. Improving social and occupational function may be a critical target due to its impacts longitudinally on self-harm and suicidality. The conditional independence of alcohol and substance use supports the need for specific interventions to target these comorbidities.

## Introduction

Mental illness affects up to two-thirds of young people prior to age 25 years (Solmi *et al.*, 2022), and the impacts they have over a lifetime are significant (Copeland *et al.*, 2015; Roberts *et al.*, 2015). The peak burden occurs during adolescence and young adulthood, even if these disorders are subthreshold or have remitted (Gore *et al.*, 2011; Jones, 2013). So, it is critical to intervene early to reduce the impact these disorders have in adulthood.

The needs of young people are complex and span multiple domains. Typical domains that are relevant to youth mental health include specific psychiatric syndromes (e.g., depression, anxiety), social and occupational function, self-harm and suicidality, alcohol and substance misuse, and physical health (Iorfino *et al.*, 2019a). For example, about one-quarter of young people presenting for care are disengaged from work or education (O’Dea *et al.*, 2014), at least one-third have suicidal ideation (Scott *et al.*, 2012) and alcohol or substance misuse is two to three times higher than the general population (Hermens *et al.*, 2013). These factors often complicate treatment approaches and may contribute to poorer outcomes (Iorfino *et al.*, 2018, 2019b). Lower rates of comorbidity are associated with better functional outcomes, while poorer trajectories of functioning tend to be associated with the presence of self-harm and suicidality, physical illness comorbidity, substance misuse and social disengagement (Iorfino *et al.*, 2022; McGrath *et al.*, 2020). Thus, youth mental healthcare requires comprehensive approaches; however, efforts to intervene among young people with emerging illness is challenging due to its heterogeneity and multidimensionality, which makes identifying targets for intervention difficult.

Little is known about the dependencies between the social, emotional and physical health factors young people present with and whether they influence each other over time. Many of these elements do not exist in isolation but are part of a dynamic, interconnected and complex system (Fried *et al.*, 2017). Understanding the functioning and dynamics of this system and its dependencies could lead to improved prediction of long-term outcomes, and inform the target and timing of interventions.

Network models have been proposed as an approach capable of modelling the complex dependencies between many factors (Koller & Friedman, 2009). Network models are often represented graphically, with factors represented by a set of nodes, and dependencies between factors represented by edges connecting pairs of nodes. A subclass of network models known as Bayesian networks (BNs) are represented by a directed acyclic graph (DAG), which has the added constraints that edges imply the direction of dependencies and cannot form closed loops. The dependency structure can then be inferred from the graph. As an example, the graph 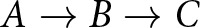 is a DAG that encodes the relationships; 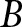 is dependent on 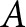, 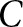 is dependent on 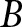, but 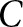 is conditionally independent of 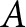 given 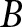. Under certain conditions (e.g., that we have all relevant factors), BNs can be used to infer causal relationships, which makes them useful to identify intervention targets.

Estimating a BN has two components. The first is estimating the structure of the network, where the structure consists of the directed edges that connect nodes. Then, given this structure, the parameters of the BN are estimated. Learning the structure of a BN is a difficult task. This is in part due to the large number of BNs that are possible, even given a small number of nodes. For example, the number of possible BNs for a 10-node network is ∼10^18^ (or greater than the number of ants on Earth). Therefore, a search over all possible graphs is computationally expensive, but has been improved significantly recently, with several procedures aiming to explore the most probable structures (Koivisto & Sood, 2004; Kuipers & Moffa, 2017; Liao *et al.*, 2019; Suter *et al.*, 2023). Exploring graph structures probabilistically has the benefit of being able to quantify the uncertainties in the graph structures.

Based on the aforementioned reasons, BNs have been used sparingly in comparison to simpler methods (e.g., correlation or partial correlation analysis) within the mental health literature. Among the studies applying BNs in mental health, most tend to focus on specific syndromes (i.e., depression [Briganti *et al.*, 2021], posttraumatic stress disorder [McNally *et al.*, 2017a], and paranoia [Bird *et al.*, 2019]) and understanding how sets of symptoms may contribute to the development, recurrence or progression of these illnesses (Hinze *et al.*, 2022; Khalifa *et al.*, 2016; Malgaroli *et al.*, 2018; McNally *et al.*, 2017b; Moffa *et al.*, 2023). Yet, from a clinical perspective, the transdiagnostic social, emotional and physical health factors are hypothesised to interact over time and contribute to poor outcomes in youth mental health. To solve this problem, we use BNs to investigate temporal dependencies; such BNs are often referred to as dynamic Bayesian networks (DBNs, Friedman *et al.*, 1998). The current study applies this approach to a longitudinal cohort of young people presenting for youth mental healthcare to infer the temporal dependencies between transdiagnostic social, emotional and physical health factors. Identifying these temporal dependencies could provide a better understanding of how changes in one domain may have direct or indirect effects on other domains over time.

## Method

The authors assert that all procedures contributing to this work comply with the ethical standards of the relevant national and institutional committees on human experimentation and with the Helsinki Declaration of 1975, as revised in 2008. All procedures involving human subjects/patients were approved by The University of Sydney Human Research Ethics Committee (2008/5453, 2012/1626), and participants (and/or guardians) gave written informed consent.

### Participants

Participants were drawn from a cohort of 6743 individuals aged 12–30 years who presented to the Brain and Mind Centre’s youth mental health clinics in two metropolitan suburbs of Sydney and recruited to a research register between June 2008 and July 2018 (Carpenter *et al.*, 2020). These clinics include early intervention primary care services (Hickie *et al.*, 2014; McGorry *et al.*, 2014) (i.e., *headspace*) as well as more specialised services, wherein they may be self-referred (walk-ins), or referred by family, friends or members of the community (including medical practitioners and universities). All participants received clinician-based case management and psychological, social and/or medical interventions as part of standard care.

### Eligibility criteria

Participants from this cohort were eligible for this study if they were also included in our longitudinal follow-up study (Carpenter *et al.*, 2020). Inclusion for this study was restricted to those with valid entries for all factors of interest at either the initial timepoint or at any two consecutives follow-up timepoints. Young people with a psychotic syndrome at initial presentation were excluded.

### Assessments

Data were extracted from clinical files, and code inputs according to proforma processes described previously (Carpenter *et al.*, 2020). The first available clinical assessment at the service is taken as the initial timepoint for each participant, and the date of this assessment is used to determine each of the follow-up timepoints. The proforma was used to record specific illness course characteristics (inter-rater reliability are reported in the supplement and cohort paper) (Carpenter *et al.*, 2020). The measures used here include (see etext 1 in supplement 1): demographics, social and occupational functioning (including the Social and Occupational Functioning Assessment Scale [SOFAS; Goldman *et al.*, 1992], and Not in Education, Employment or Training as a measure of participation and engagement with education or work), mental disorder diagnoses, clinical stage, at-risk mental states, self-harm, suicidal thoughts and behaviours, alcohol and substance use, physical health comorbidities, personal mental illness history and treatment utilisation. All factors were categorical. Diagnoses were categorised as either subthreshold, full-threshold or none. SOFAS was treated as binary with ‘good’ functioning represented as SOFAS >70 and ‘poor’ otherwise (based on current accepted clinical cut-offs). Age was also treated as a binary factor with age >18 years as the cut-point (differentiating school-aged and non-school aged young people). All other factors were entered in a binary format.

### Statistical analyses

We used a DBN (Friedman *et al.*, 1998) for our primary analysis. Our DBN starts with a BN that represents the dependencies between factors that were recorded at first presentation, referred to as an ‘initial network’. To estimate the initial network, we used all individuals that had valid entries across all factors of interest. Following the initial network, a ‘transition network’ is developed, which represents the dependencies between consecutive timepoints. We assume that the transition network is the same across any two consecutive timepoints. Thus, we used any individual in our data that had valid entries across all factors at consecutive timepoints. The combination of the initial and transition networks defines a DBN across all timepoints in our data.

We also investigated two sets of assumptions for the dependencies at the subsequent timepoints in our transition network. Firstly, given a transition network for consecutive timepoints (*t*, *t* + 1), we assume that factors measured at timepoint *t* + 1 are conditionally independent given factors at the previous timepoint *t* (i.e., temporal dependencies). In the secondary analysis, we allow for factors at *t* + 1 to be dependent on other factors at *t* + 1 (i.e., temporal & contemporaneous dependencies).

Estimation of network structures was addressed within a Bayesian framework; thus, inference was via the posterior distribution for each network. We investigate the posterior probability distribution for a BN 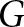
given the data 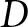, which is 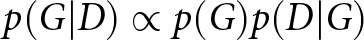. Bayesian inference provides a mechanism to update our prior knowledge of the problem, quantified as 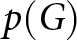, with the likelihood of the data, quantified as 
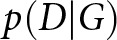
. Our prior knowledge is used to remove unrealistic edges from the graphs (see etext 2 in supplement 1).

An advantage of framing our problem in terms of Bayesian inference is that we can use algorithms to generate samples of BNs proportional to the posterior distribution. Posterior samples can then be used to estimate quantities of interest. For example, the probability that an edge exists in the network is quantified as the number of times the edge occurs in our samples divided by the number of samples. Posterior sampling was achieved using the Partition Markov chain Monte Carlo scheme available in the R language (Suter *et al.*, 2023).

For plotting and calculation of edge probabilities, we first convert each BN in our posterior sample to a completed partially directed acyclic graph (CPDAG). The CPDAG is a graph that includes edges in both directions where either direction leads to the same posterior probability. After this conversion, we calculate the edge probability in each direction.

We also estimated the direct and indirect effects between factors (Kuipers *et al.*, 2019; Moffa *et al.*, 2017). We quantified these effects, averaged over our posterior sample of DAGs, from factor A to B as the relative risk (RR), where 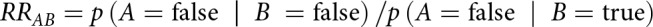. Below we report the expected value (µ) and 95% highest density credible interval (CI, the Bayesian counterpart of confidence intervals) (Makowski *et al.*, 2019). To calculate 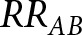 for the multiple category diagnosis factors, we assign true to full- and sub-threshold and false otherwise. These calculations required an estimate of the conditional probabilities between pairs of conditionally dependent nodes for each BNs in our posterior samples, which is outlined in etext 4 in Supplement 1.

## Results

### Sample characteristics

The cohort was comprised of 2663 individuals (61% female) with a mean age of 18.55 ± 3.69 years at baseline. Table 1 shows the characteristics of this cohort.

**Table 1.** Baseline demographic and clinical characteristics of longitudinal youth cohort

Timepoint = 0	Total	Male	Female
*N* (%)	2663 (100)	1038 (39.0)	1625 (61.0)
**Demographics**			
Age, mean (SD)	18.55 (3.69)	18.59 (3.80)	18.53 (3.62)
**Social and occupational function**			
SOFAS score, mean (SD)	62.74 (9.07)	61.18 (9.17)	63.73 (8.86)
**At-risk mental states**, *N* (%)			
Manic-like experiences	424 (15.9)	133 (12.8)	291 (17.9)
Psychosis-like experiences	394 (14.8)	172 (16.6)	222 (13.7)
Circadian disturbance	391 (14.7)	136 (13.1)	255 (15.7)
**Clinical presentation**, *N* (%)			
Depression			
Subthreshold	1218 (45.7)	429 (41.3)	789 (48.6)
Full-threshold	847 (31.8)	317 (30.5)	530 (32.6)
Anxiety			
Subthreshold	955 (35.9)	350 (33.7)	605 (37.2)
Full-threshold	698 (26.2)	257 (24.8)	441 (27.1)
Bipolar			
Subthreshold	161 (6.0)	44 (4.2)	117 (7.2)
Full-threshold	114 (4.3)	39 (3.8)	75 (4.6)
Substance-related or addictive disorder			
Subthreshold	103 (3.9)	54 (5.2)	49 (3.0)
Full-threshold	141 (5.3)	82 (7.9)	59 (3.6)
**History of mental illness in childhood**, *N* (%)			
Anxiety	68 (2.6)	18 (1.7)	50 (3.1)
Depression	41 (1.5)	16 (1.5)	25 (1.5)
Neuro-developmental	261 (9.8)	181 (17.4)	80 (4.9)
**Family history of mental illness**, *N* (%)			
Family history of depression	969 (36.4)	333 (32.1)	636 (39.1)
Family history of anxiety	409 (15.4)	160 (15.4)	249 (15.3)
Family history of bipolar	209 (7.8)	65 (6.3)	144 (8.9)
Family history of psychosis	109 (4.1)	36 (3.5)	73 (4.5)
Family history of addiction	341 (12.8)	123 (11.8)	218 (13.4)
Family history of suicide	45 (1.7)	19 (1.8)	26 (1.6)
**Physical health comorbidities**, *N* (%)			
Any major physical illness	431 (16.2)	144 (13.9)	287 (17.7)
**Self-harm and suicidal thoughts and behaviours**, *N* (%)			
Self-harm	1032 (38.8)	249 (24.0)	783 (48.2)
Suicide ideation	1210 (45.4)	420 (40.5)	790 (48.6)
Suicide attempt	380 (14.3)	119 (11.5)	261 (16.1)
**Alcohol and substance use**, *N* (%)			
Alcohol use	1644 (61.7)	641 (61.8)	1003 (61.7)
Tobacco use	976 (36.7)	393 (37.9)	583 (35.9)
Cannabis use	1001 (37.6)	439 (42.3)	562 (34.6)
Other drug use	595 (22.3)	244 (23.5)	351 (21.6)

Descriptive statistics are shown if they appeared in either the initial or transition networks

SOFAS = Social and Occupational Functional Assessment Scale. SOFAS scores at baseline were missing for 14 individuals.

### Initial network structure – dependencies at the initial timepoint

**Figure 1.**
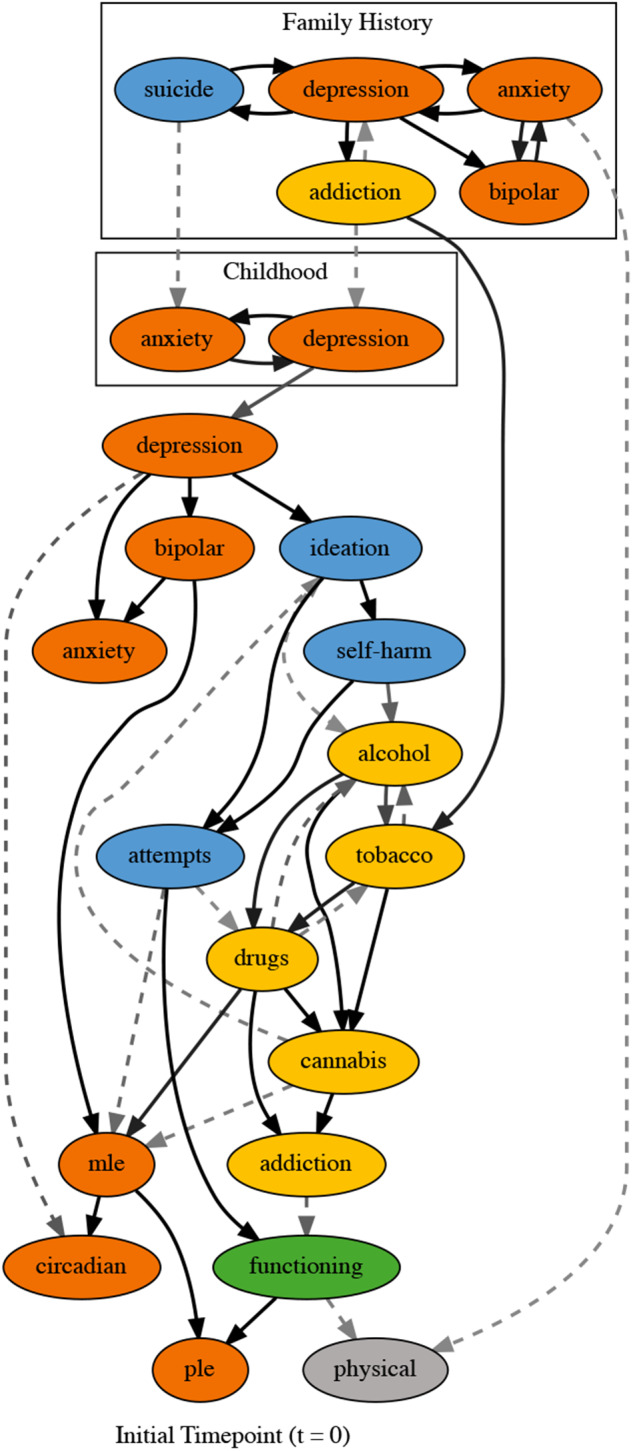
Summarised structure for the initial network, which relates factors at the initial timepoint (*t* = 0), along with family history and childhood onset conditions. This is not a single DAG, but rather a summary of all DAGs in the posterior sample. We show edges that occurred with probability > 0.1. Solid lines represent edges that appeared in the completed partially directed acyclic graph (CPDAG) maximum a posteriori (MAP) estimate. Edge transparency decreases with probability. Dashed lines represent edges that don’t appear in the MAP but are in >10% of sampled DAGs. Node colours represent different domains: social and occupational function (green), self-harm and suicidality (blue), alcohol and substance use (yellow), physical health (grey) and psychiatric syndromes (orange).

Figure 1 summarises the conditional dependencies between factors at initial presentation (*t* = 0). Across-domain edges that appear with *p* > 0.99 include depression to suicidal ideation, suicide attempts to functioning, other drug use to manic-like experiences (MLEs), functioning to psychosis-like experiences (PLEs) and MLEs to PLEs. Several edges across domains occur with lower probability including self-harm to alcohol (*p* = 0.41), addiction to function (*p* = 0.41), suicide attempts to MLEs (*p* = 0.34), cannabis to MLEs (*p* = 0.21) and cannabis to suicidal ideation (*p* = 0.22) (see etext 5 in Supplement 1 for other probable structures).

### *Transition network structure – temporal dependencies between factors over time (*t > t *+ 1)*

**Figure 2.**
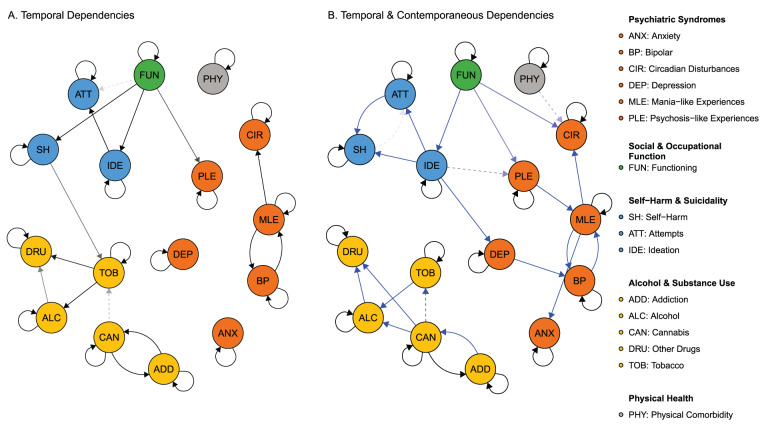
Summarised structure for the transition network (*t* → *t* + 1) with (Panel A), and without (Panel B) the assumption that factors at *t* + 1 are conditionally independent. This is not a single DAG, but rather a summary of all DAGs in the posterior sample. We show edges that occur with probability >0.1. Solid edges represent those that appeared in the completed partially directed acyclic graph (CPDAG) maximum a posteriori (MAP) estimate. Edge transparency decreases with probability and blue lines show contemporaneous edges. Dashed lines represent edges that don’t appear in the MAP but are in >10% of sampled DAGs. Node colours represent different domains: social and occupational function (green), self-harm and suicidality (blue), alcohol and substance use (yellow), physical health (grey) and psychiatric syndromes (orange).

The transition network from *t* to *t* + 1 assuming conditional independence of factors at *t* + 1 is shown in Fig. 2a. Same factor edges across timepoints (i.e., 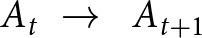) were observed for all factors with *p* > 0.99. Across domain relationships were found for social and occupational functioning to self-harm (*p* = 0.97), suicidal ideation (*p* > 0.99) and PLEs (*p* = 0.71). We also found an edge from self-harm to tobacco use (*p* = 0.59). Many within domain relationships were observed with probability > 0.95. These relationships included tobacco use to alcohol use and suicide ideation to suicide attempts. Some within domain relationships were observed in both directions.

A second transition network that accounted for dependencies at *t* + 1 is shown in Fig. 2b. This network exhibits similar same factor and within domain relationships as shown in Fig. 2a. However, a more complex pathway was identified for social and occupational functioning, self-harm and suicidality and alcohol and substance use, leading to syndromes and at-risk states. Subsequent analysis throughout this paper will use this model.

### Direct and indirect temporal effects

**Figure 3.**
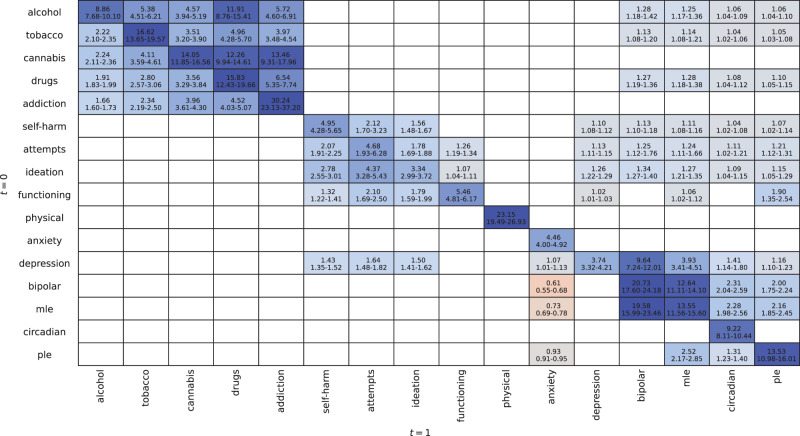
Relative risks (RR) between factors from timepoint 0 to timepoint 1 (i.e., rows to columns) allowing for dependencies of factors at *t* + 1. Cells contain the RR for the poor outcome occurring at timepoint 1 given the change in another factor at timepoint 0. The RR mean (µ) and 95% highest density credible interval (CI) are presented. Values are only shown where the CI does not contain 1.

The magnitude and sign of the temporal dependencies identified by the transition network are presented in Figure 3 (i.e., direct and indirect effects from timepoint 0 to 1). For most factors, the greatest effects were due to the same factor at the previous timepoint. Psychiatric syndromes typically had the strongest effects on that state at the next timepoint with addiction (RR, 30.24; CI, 23.13–37.20) having the highest probability of persistence, while anxiety (RR, 4.46; CI, 4.00–4.92) had the lowest probability of persistence. A strong reciprocal effect was also observed for the at-risk state MLE with bipolar disorder. PLE affected MLE (RR, 2.52; CI, 2.17–2.85) and circadian disturbances (RR, 1.31; CI, 1.23–1.40).

For the suicidality domain, suicide ideation had the largest effects within domain with effects on attempts (RR, 4.37; CI, 3.28–5.43) and self-harm (RR, 2.78; CI, 2.55–3.01). Outside of this domain, social and occupational function had the largest effect on suicidality, with effects on ideation (RR, 1.79, CI, 1.59–1.99), attempts (RR, 2.10, CI, 1.69–2.50) and self-harm (RR, 1.32, CI, 1.22–1.41). Depression was the only other factor to have a larger effect on self-harm (RR, 1.43; CI, 1.35–1.52).

Alcohol and substance use primary effects were within its own domain, with no other domains having any effect on these factors. The within domain effects showed increasing severity of substance use (e.g., alcohol and tobacco to cannabis use). The largest effect was from alcohol use to drug use (RR, 11.91; CI, 8.76–15.41), while addictive disorder was mostly affected by cannabis use (RR, 13.46; CI, 9.31-17.96) and drug use (RR, 6.54; 5.35-7.74). Bipolar disorder, MLE, circadian disturbances and PLE were affected by alcohol and substance use.

## Discussion

This study used BNs to identify the temporal dependencies between multiple domains which provides insights into their role in the course of illness among those engaged in youth mental healthcare. The initial network shows potential developmental trajectories of disorder among young people. The transition network identified the specific role each of these factors has in the course of illness. Over time, social and occupational function had a direct effect on suicide ideation and self-harm, and indirect effects on suicide attempts. Suicide ideation had a direct effect on suicide attempts and self-harm. Alcohol and substance use effects tended to be within that domain, and physical health, psychiatric syndromes were similarly independent whereby all effects remained within each domain over time. This work helps us understand the complex dynamics between multiple transdiagnostic social, emotional and physical health factors, which could improve clinical decision-making regarding targets for intervention based on anticipated direct and indirect effects on outcomes.

The initial network describes the dependencies between domains observed at entry into care. This work is consistent with evidence for the role of genetic and familial vulnerabilities on the emergence of depression during adolescence and young adulthood (Vandeleur *et al.*, 2012). Childhood disorders preceding adolescent depression was associated with three distinct pathways or illness trajectories: (1) anxiety disorder; (2) bipolar disorder, MLE, circadian disturbances and PLE; and (3) self-harm and suicidality to alcohol and substance use or functioning. These pathways support hypotheses speculating differential illness types associated with early-stage mood disorders, namely those related to a manic-like, circadian rhythm dysfunction type (Crouse *et al.*, 2021), and an anxious, hyperarousal and stress reactivity type (Hickie *et al.*, 2019). The distinct trajectory for self-harm and suicidality, alcohol and substance use, and functioning suggest that these factors may not be directly associated with illness progression and have a somewhat independent path whereby their emergence is conditional on other factors. Similarly, the progression to more severe substance use was dependent on a family history of addiction (Khurana *et al.*, 2015) and suggests the need for specific secondary prevention among those with this history or engaging in early alcohol or tobacco use behaviours (Hoffmann & Cerbone, 2002; SAMHSA, 2013). Particularly, given the effects such substances have on brain development which is consistent with our finding that MLE, PLE and potentially suicidal ideation are downstream of these factors (Winters & Arria, 2011).

Longitudinally, we see quite a distinct pattern of temporal effects between impairment and self-harm and suicidality when compared to the initial network. Poor social and occupational functioning was associated with a higher probability of suicide ideation or self-harm at the next timepoint (Iorfino *et al.*, 2020). So, while decreases in function due to suicide ideation may precede seeking mental healthcare, the persistence of functional impairment for those engaged in care may have an on-going effect on suicide ideation, which itself had a direct effect on suicide attempts. This would indicate that improving function may be an important intervention target for keeping people safe and reducing these risks. Further to this are the potential effects of functional impairment on PLEs which were also observed in these networks. This is critical given the increased rate of PLEs in those presenting to youth mental health services and the future risks they confer (Capon *et al.*, 2023; Yung *et al.*, 2008). Previous research has shown the importance of the direct impacts of improving social and occupational functioning through individual placement support (Heffernan & Pilkington, 2011; Killackey *et al.*, 2008; Michon *et al.*, 2014); however, indirect effects of these interventions could be expected on suicide ideation and self-harm (McGorry *et al.*, 2022).

The conditional independence of alcohol and substance use factors in the transition network has important implications for intervention and secondary prevention. Despite major comorbidity between mental disorders and alcohol and substance use, our models identified no temporal relationship between these domains. This reiterates that treatment for comorbid mental disorders and alcohol or substance use cannot rely on the assumption that resolving the primary disorder would be sufficient to address alcohol or substance use (Brady *et al.*, 2007; Pettinati *et al.*, 2013). Specific interventions that directly address these behaviours, such as cognitive behaviour therapy and motivational interviewing (Riper *et al.*, 2014) or relevant pharmacological treatments, are needed for effective primary and secondary prevention of alcohol and substance use disorders which tend to be undertreated in mental healthcare settings (Mintz *et al.*, 2021). This would require effective care coordination of multiple interventions to facilitate the delivery and timing of these interventions in a way that optimises engagement and adherence (Gaudiano *et al.*, 2011; Iorfino *et al.*, 2021; Mann *et al.*, 2017). Yet, such targeted and coordinated interventions may have major cascading effects across other domains since we observed causal effects for alcohol and substance use on bipolar disorder, MLE, circadian disturbances and PLEs.

Finally, the interpretations of dependencies are made easier using BNs compared to other associational analyses (e.g., correlation analysis, see etext 7 in supplementary 1). This is particularly true for the contemporaneous timepoint dependencies as seen in the initial network which provides insight into the development of mental ill health prior to care, and the transition network allowing for the inference of directionality while controlling for changes in factors between timepoints. For example, the transition network allowing for dependencies at *t* + 1 suggests that social and occupational function, suicidal ideation and suicidal attempts change together. An interpretation of this is that the risk of suicide attempts at the next timepoint is related to social and occupational functioning and suicidal ideation between timepoints. This BN approach allows for richer insights into these relationships that aligns more closely with the necessary evidence required for guiding clinical decisions about specific treatment targets.

### Limitations

This cohort is a selected subset of a larger cohort of young people (aged 12–30 years) that participated in a range of assessments (39.5%, 2663/6743). As such, this sample may not be representative of all help-seeking individuals, with a potential bias towards those who continue to engage with services. Another limitation is that data were extracted from clinical records when individuals attended clinical services, rather than via structured follow-up assessments. This has resulted in individuals who received clinical care with differing frequency and duration. Individuals may also have periods where they do not attend services but reappear later in the data. To limit the effects of this issue, we used any two consecutive timepoints to infer the transition network. This allowed us to study the maximum number of consecutive transitions in our data, although with the added assumption that the data are missing at random. Selection bias can also have effects on causal inference, which can be remedied by adding nodes that account for such biases (Nohr & Liew, 2018), although this often requires assumptions about the underlying population, and thus we do not attempt to correct for it in this analysis. Furthermore, as data were extracted from clinical records, the consistency in record keeping may differ. This may have led to the omission of factors, which could limit our ability to infer dependencies between factors. While this may be an issue, we note that the data entry and collection was performed by trained individuals, focuses on clinically relevant and available information and has been shown to have acceptable interrater reliability (Carpenter *et al.*, 2020). The lack of direct effects observed for psychiatric syndromes and physical health domains could be partially explained by the lack of change observed in these variables over time. These were simple diagnostic variables that were subject to lower rates of change but were included here because they are expected to be associated with many outcomes of interest. Future studies may benefit from using more dynamic measures of these domains to investigate their effects over time.

While BNs represent the dependencies between factors in our data, we must be cautious to interpret the causal nature of these relationships. Inferring causality from BNs can only be justified under certain conditions. In particular, BNs assume acyclicity, which may not be justified, particularly for the initial network where the current state of an individual will have developed over a significant period of time, thus allowing feedback loops to develop. In such circumstances, we are likely only inferring the primary pathways by which individuals develop (Moffa *et al.*, 2017). Furthermore, we must assume that we are not missing any confounding or colliding factors (Spirtes *et al.*, 2012) and that edge weights between factors do not cancel out in such a way that we would be unable to infer them from our data. There are likely other clinical, physical or social factors relevant to the BNs which are missing from this analysis and yet may be an important predictor in the network. We also estimated the BN structure for the entire cohort (not the individual level); however, it is plausible that different network structures could be used to describe different subgroups of people whereby different processes are at play.

## Conclusions

A major challenge for youth mental healthcare is to provide personalised care that improves a range of social, emotional and physical health outcomes. This study identified temporal dependencies between multiple domains, which has causal interpretations, and thus sheds light on intervention targets such as social and occupational function which could have widespread effects. In contrast, the effects for alcohol and substance use were independent of other domains over time and emphasise the importance of specific early intervention and secondary prevention for these comorbidities.

## Supporting information

Iorfino et al. supplementary material Iorfino et al. supplementary material

## Data Availability

The data that support the findings of this study are available from the corresponding author, FI, upon reasonable request. Drs Hickie and Iorfino had full access to all the data in the study and take responsibility for the integrity of the data and the accuracy of the data analysis.

## Supplementary Material

The supplementary material for this article can be found at https://doi.org/10.1017/S2045796023000616.
