# Supplementary material for: The temporal dependencies between social, emotional and physical health factors in young people receiving mental healthcare: a dynamic Bayesian network analysis
Source: Epidemiol Psychiatr Sci. 2023 Sep 8;32:e56. doi: 10.1017/S2045796023000616 (PMC10539737; doi:10.1017/S2045796023000616)
Supplement: Iorfino et al. supplementary material [file S2045796023000616sup001.docx]

**Supplementary file 1**

**etext 1.** Further information about the variables used in this study

**etext 2.** Edges removed from the prior distribution for the graph structure

**table 1.** Removed edges within a timepoint from our prior distribution

**etext 3.** Reliability of our posterior samples

**table 2.** Expected log posterior predictive density for the transitions from t to t+1 for each model.

**etext 4.** Estimating conditional probability parameters and causal effects

**etext 5.** The complexity of the joint probability distribution of edges in the initial networ**figure 1.** Conditional Initial network structure.**etext 6.** Description of individuals used per timepoint

**table 4.** The distribution of transitions used per individual.

**table 3.** The number (N) of individuals and the typical time difference per timepoint

**etext 7.** Correlation and partial correlation analysis

**Figure 2.** The correlation and partial correlation matrices for factors at the initial observation.

**Figure 3.** The correlation and partial correlation matrices for factors across timepoints

This supplementary material has been provided by the authors to give readers additional information about their work.

**etext 1.** **Further information about the variables used in this study**

The content presented in etext 1 is adapted from supplementary material published by Iorfino et al. (2019) (1).

The clinical notes for all study participants were manually read and assessed by a team of trained clinical researchers. Data was then extracted from these clinical notes by the clinical researchers and entered into the proforma as required. All clinical notes are generated by the study participants’ treating clinician/s as part of their standard care.

**Demographics**

Biological sex, and age. Current engagement in part- or full-time education or employment to determine Not in Education, Employment, or Training (NEET) status. NEET is assigned if there was no full- or part-time education, employment, training, or volunteer work.

**Social and Occupational Functioning**

The Social and Occupational Functioning Assessment Scale (2) (SOFAS) is a clinician-rated measure that assesses functioning on a 0–100 scale, with lower scores suggesting functional impairment. The instructions emphasise that the rater should avoid confounding the rating with clinical symptoms (2–4). A SOFAS score of below 70 is considered to be clinically-significant impairment (5).

**Mental Disorder Diagnoses**

Mental disorder diagnoses at each time point are classified according to DSM-5 criteria (6) and specified as either full- or sub-threshold. Diagnoses are also labelled as either primary, secondary, or tertiary based on judgement of which was the dominant presenting problem at that time point.

Mental disorder diagnosis is determined solely by the symptomology &/or diagnosis reported and recorded by the treating clinician/s as presented in the clinical notes of each study participant. Based on the information provided within these clinical notes, researchers determined whether DSM-5 criteria were met for a specific disorder at that time point. If symptomology recorded in the clinical notes indicated only some, but not all criteria being met for a specific disorder, then a sub-threshold classification was recorded. If symptomology indicated full DSM-5 criteria were met for that time point, then a full-threshold classification was recorded.

As per diagnosis, medication is also obtained from a review of the clinical notes as generated by the study participants’ clinician/s. A certain medication is recorded if the clinical notes indicate that the study participant took that particular class of medication within the specified timeframe.

**Clinical Stage**

Information about the course of illness is also used to assign a clinical stage at each time point according to a previously established model (7–9) and as described in the introduction. While stages 3 and 4 are also specified elsewhere for recurrent, persistent, and chronic illness courses, stage 2 is our proposed cut-point for more persistent disorders requiring more specific and intensive clinical care and treatment (7–9). With regards to longitudinal assessment, and consistent with other clinical staging models in medicine (e.g. oncology), while an individual may experience clinical remission at any stage, once they reach a certain point on the illness continuum they cannot go back to an earlier stage.

**At-risk Mental States**

Clusters of symptoms that have been previously indicated as risk factors for progression to more severe mental disorders (10–15) are recorded in all individuals regardless of diagnosis. This includes psychotic-like experiences (the presence of any psychotic symptoms including: perceptual abnormalities, bizarre ideas, disorganised speech, etc), manic-like experiences (the presence of any manic/hypomanic symptoms including: abnormally elevated mood or irritability; increased motor activity, speech, or sexual interest, etc), and circadian disturbance (the presence of significant disruption in sleep-wake or circadian cycles including the presence of a severe sleep-wake disorder or chronic fatigue). The presence or absence of these clusters of symptoms is determined solely by the symptomology reported and recorded by the treating clinician/s as presented in the clinical notes of each study participant. Similarly, the distinction between psychotic-like and manic-like symptoms is judged within the context of the clinical notes.

The threshold for mania like experiences and psychotic like experiences in this study is low. Conversely, the threshold for circadian disturbance in this study is high. More specifically, these experiences are rated based on their presence or absence and the nature (e.g. type, severity, frequency) of these experiences, and so stage 1a and stage 1b MLEs and PLEs are not necessarily different, but in some cases may differ in nature. The presence of these symptoms does not necessarily mean the participant currently has / or will go on to develop a serious mental health disorder. It is simply one of many risk factors that may exist. Moreover, the presence or absence of these symptoms do not, in and of themselves, determine the staging of a participant.

**Self-harm and Suicidal Thoughts and Behaviours**

The presence of suicidal ideation, suicide attempts, and self-harm is recorded. A suicide attempt is recorded when a young person has taken steps to take their own life. If an individual harms themselves via cutting, hitting themselves, burning themselves, or scratching with the intention to self-harm only and not to take their life, then this is included as self-harm and not a suicide attempt.

**Physical Health Comorbidities**

Any major physical illness is recorded.

**Personal Mental Illness History**

Known childhood-onset disorders (i.e. with clear onset prior to 12 years old) are recorded in addition to current diagnoses. Family history of a mental health disorder is ascertained via the treating clinician’s assessment with the client. Please note, family history is only recorded when the client has reported a mental health diagnosis of a first degree relative. Moreover, family history is only recorded if the client reported that the first-degree family member has a current of past diagnosed mental health disorder. Symptoms only, but no diagnosis, is not enough to meet criteria for this category.

**Treatment Utilisation**

Exposure to classes of medication (antidepressant, antipsychotic, mood stabiliser, or stimulant medication), and hospitalisation overnight or longer due to a mental health problem are recorded.

etext 2. **Edges removed from the prior distribution for the graph structure**

We removed the parent to child edges within a timepoint that are shown in Table 1. We also removed any edges that had a direction backwards in time.

| **Parent** | **Child** |
| --- | --- |
| Depression | MLE |
| Depression | Bipolar |
| Suicide Attempts | Suicide ideation |

**Table 1:** Removed edges within a timepoint from our prior distribution.

**etext 3. Reliability of our posterior samples**

The reliability of our posterior samples was investigated for each network using several methods. Firstly, we ensured that our posterior samples included the maximum scoring DAG found from running the iterative MCMC (16) optimisation algorithm several times starting from random positions. We also checked that the edge probabilities were consistent across multiple runs of Partition MCMC. The mean difference in edge probabilities for all networks across multiple runs was < 0.01.

The number of iterations for the MCMC sampler was decided on a trial-and-error basis. The initial network and the transition network that included the contemporaneous dependencies were ran for 50N^2^log(V), where V is equal to the number of nodes that could be a child node (e.g. not including nodes at t for the transition). This dependency is a slight modification to the one recommended in the BiDAG package, resulting in a greater number of iterations. We retained 50k iterations for the initial network and transition networks that were used for the final analysis. The transition matrix with independent dependencies at t+1 was only ran for 10k iterations where we retained all samples, as this sampling is much simpler, requiring only random draws of the DAGs from already defined partitions, thus requiring no partition or node moves.

We performed posterior predictive tests to measure the predictive performance for the transition networks. The models were split into training and test sets using a 10-fold cross-validation procedure. We then calculated the out of sample expected log posterior predictive probability (ELPD)(17), for all variables at t+1 given all variables at t summed across all t to t+1 transitions in our data. That is, $ELPD=\sum_{k=1}^{10} \sum_{\{m\in M_{k}\}} log\left( \frac{1}{S}\sum_{s=1}^{S} \prod_{i=1}^{n} p(d_{im}|\theta^{ks}, X^{ks} ) \right)$ where $d_{im}$ is the observed value of the i-th factor for individual m that is in $M_{k}$ which is the set of test individuals for cross-validation split k. Then $X^{ks}$ represents the parent set and $\theta^{ks}$ represents the parameters for the $s$-th simulated draw using the $k$-th training set. We also report the uncertainty in the ELPD as $\Delta ELPD=\sqrt{\sum_{k=1}^{10} \sum_{\{m\in M_{k}\}} {Var}_{s=1}^{S} \left( \log\prod_{i=1}^{n} p(d_{im}|\theta^{ks},X_{Y_{i}}^{ks} ) \right)}$ . Taking the product of the factors at t+1 treats them as independent, thus we’re testing the marginal rather than joint predictive accuracy. Our models were compared to a naïve model, that assumed factors at t+1 were only dependent on the same variable at t. The ELPD calculations are shown in in Table 2. Both models performed better than the naïve model, with the model assuming independence at time t+1 performing best. It’s unsurprising that the model assuming independence at t+1 has a greater ELPD due to testing marginal rather than joint predictive accuracy.

| **Model** | **ELPD** |
| --- | --- |
| Independent at t + 1 | -23699.0±28.4 |
| Dependent at t + 1 | -23767.8±28.6 |
| Naïve | -24526.1±7.8 |

**Table 2:** Expected log posterior predictive density for the transitions from t to t+1 for each model.

**etext 4. Estimating conditional probability parameters and causal effects**

The causal effects require an estimation of the graph parameters. To do this, we estimate the conditional probability for each factor given its parent states. We need to estimate the probability distribution for a factor A = true with a given graph structure and parent state $\text{X}=\{X_{0}=x_{0}, X_{1}=x_{1}, \ldots, X_{p}=x_{p}\}$ where $x_{i} \epsilon\{\text{true}, \text{false}\}$. The posterior probability is then $p\left( A=\text{true } \right|\text{ }\text{D}\text{, }\text{X }\text{)}\text{ = p(A = true | }\text{X}\text{)p(}\text{D}\text{ | A=true, }\text{X}\text{)}$, where the data is given by the counts of each state given the condition $\text{X}$, that is $\boldsymbol{D}=\{n\left( A=\mathrm{true} | \boldsymbol{X} \right), n\left( A=\mathrm{false} | \boldsymbol{X} \right)\}$. We assume a beta prior for all factors that are independent of the parent set with parameters $\alpha=\beta=1$ and a binomial likelihood function. The posterior distribution is then a beta distribution with updated parameters $\alpha_{A\boldsymbol{X}}= \alpha+n\left( A=\text{true} \right| \boldsymbol{X)}$and $\beta_{A\boldsymbol{X}}= \beta+n\left( A=\text{false} \right| \boldsymbol{X})$. The conditional probabilities between factors that are not directly connected are calculated by using the graph structure.

The above calculations are then used to calculate the downstream relative risk ratio which we refer to as the causal effect. The causal effect of B on A is defined as $\text{RR}_{AB}=p\left( A=\text{true} \right|B=\text{true})/p(A=\text{true}|B=\text{false})$. This calculation is marginalized over the nuisance parameters and the graph structure. To marginalise over the graph structure we calculate $\text{RR}_{AB}$ 10^4^ times where we sample the graph structure each time from our posterior sample.

The computational implementation of the above calculations were implemented in R. The gRain library (18) was used to formulate and parameterise the network structure, and then calculate downstream probabilities.

**etext 5.** **The complexity of the joint probability distribution of edges in the initial network**

The posterior distribution for the initial network includes a significant number of possible DAGs. We summarize these graph structures in Figure 1 showing the MAP CPDAG and the marginalized edge probabilities above a probability cutoff value. However, the joint probability distribution for the edges is complex. Here we show that conditioning on the directionality between self-harm and suicidality with alcohol and substance misuse yields distinctly different structures (see Figure 1). Conditioning the summarised structures on any edge from the self-harm and suicidality domain to the alcohol and substance misuse domain (p = 0.62) shows that the alcohol and substance misuse domain is directly dependent on a combination of family history of addiction as well as self-harm and suicidality. Whereas conditioning the summarised structures on there being an edge from the alcohol and substance misuse domain to the self-harm and suicidality domain (p = 0.30) shows that alcohol and substance misuse is directly dependent on a family history of addiction, and that suicidal ideation is directly dependent on alcohol and substance misuse and depression. As such, while the probability that these domains are dependent is high (p = 0.89), the dependency structure, and therefore, the potential causal pathways that they describe are quite different. For this reason, we focus our attention on high probability pathways.

**Figure 1.** Initial network structure conditioned on; (Panel A) including at least one edge from the self-harm and suicidality domain to the alcohol and substance misuse domain, and (Panel B) including one edge from the alcohol and substance misuse domain to the self-harm and suicidality domain.

**etext 6.** **Description of individuals used per timepoint**

In Table 3 we show the number of individuals and their typical time difference per timepoint. The average number of pairwise observations used to infer the transition network was 2.34 (CI: 1-5).

**Table 3. The number (N) of individuals and the typical time difference per timepoint.**

| **Network** | **Timepoint (t)** | **N** | **Time difference in days, mean (SD)** |
| --- | --- | --- | --- |
| Initial | 0 | 2628 |  |
| Transition | Total | 4546 | 165 (112) |
|  | 0 | 1701 | 93 (17) |
|  | 1 | 1045 | 94 (24) |
|  | 2 | 747 | 181 (47) |
|  | 3 | 466 | 334 (78) |
|  | 4 | 259 | 347 (87) |
|  | 5 | 170 | 354 (86) |
|  | 6 | 120 | 336 (80) |
|  | 7 | 38 | 335 (121) |

**etext 7. Correlation and partial correlation analysis**We also explored correlation and partial correlation methods to understand the dependencies between factors over time. These analyses were completed in R using the psych(19) and ppcor(20) libraries. We assumed spearman correlations and only show values where p-value > 0.05/m where m was the number of tests, thus applying a Bonferroni correction.

In Figure 2 we show the correlation analyses for factors that were observed at the initial presentation of individuals within clinical care. Figure 3 shows the correlation analyses for factors across timepoints. The analysis between timepoints calculates the partial correlation of a factor $A_{t}$ with a factor $B_{t+1}$ while only conditioning on other factors at t, thus making it similar to the Bayesian network analysis assuming independence of factors at t+1, which is shown in Figure 2A of the manuscript.

Qualitatively, these analyses show some similar results to those found by the Bayesian network analysis. The within timepoint and with domain factors (e.g. alcohol and tobacco or suicidal ideation and suicidal attempts) have the strongest association. We also see that functioning has an association with suicidal thoughts and behaviours domain across timepoints.

However, these correlation based analyses have differences to the Bayesian network analysis. Correlations correspond to marginal effects and usually lead to a ‘full’ matrix with many statistically significant dependencies. Partial correlations correspond to the correlation between two factors after conditioning on the remaining factors, which leads to a sparser matrix. Often these partial correlation matrices are shown as undirected networks (21). Bayesian networks are an advancement on these techniques, as they can be used to find conditional directed dependencies, due to fitting the network making assumptions about acyclicity. As such, Bayesian networks typically have more explanatory power than correlation or partial correlation analyses(22), which is the primary motivating factor for their use in the final analysis.

Figure 2. The correlation (top) and partial correlation (bottom) matrices for factors at the initial observation. These include family history (‘f.’ prefix), childhood (‘ch.’ prefix), and other factors observed at entry to car. The cells show the spearman correlation coefficient values.

**Figure 3.** The correlation (top) and partial correlation (bottom) matrices for factors across timepoints. The cells show the spearman correlation coefficient values.

**References**

1. Iorfino F, Scott EM, Carpenter JS, Cross SP, Hermens DF, Killedar M, et al. Clinical Stage Transitions in Persons Aged 12 to 25 Years Presenting to Early Intervention Mental Health Services With Anxiety, Mood, and Psychotic Disorders. JAMA Psychiatry. 2019 Nov 1;76(11):1167.

2. Goldman HH, Skodal AE, Lave TR. Revising axis V for DSM-IV: a review of measures of social functioning. Am J Psychiatry. 1992 Sep;149(9):1148–56.

3. Hilsenroth MJ, Ackerman SJ, Blagys MD, Baumann BD, Baity MR, Smith SR, et al. Reliability and Validity of DSM-IV Axis V. Am J Psychiatry. 2000 Nov;157(11):1858–63.

4. Hay P, Katsikitis M, Begg J, Da Costa J, Blumenfeld N. A Two-Year Follow-Up Study and Prospective Evaluation of the *DSM-IV* Axis V. Psychiatr Serv. 2003 Jul;54(7):1028–30.

5. Rickwood DJ, Mazzer KR, Telford NR, Parker AG, Tanti CJ, McGorry PD. Changes in psychological distress and psychosocial functioning in young people visiting headspace centres for mental health problems. Med J Aust. 2015 Jun;202(10):537–42.

6. American Psychiatric Association. American Psychiatric Association: Diagnostic and statistical manual of mental disorders. 5th Edition. Arlington, VA; 2013.

7. Hickie IB, Scott EM, Hermens DF, Naismith SL, Guastella AJ, Kaur M, et al. Applying clinical staging to young people who present for mental health care: Applying clinical staging in young people. Early Interv Psychiatry. 2013 Feb;7(1):31–43.

8. McGorry PD, Hickie IB, Yung AR, Pantelis C, Jackson HJ. Clinical Staging of Psychiatric Disorders: A Heuristic Framework for Choosing Earlier, Safer and more Effective Interventions. Aust N Z J Psychiatry. 2006 Aug;40(8):616–22.

9. McGorry PD, Purcell R, Hickie IB, Yung AR, Pantelis C, Jackson HJ. Clinical staging: a heuristic model for psychiatry and youth mental health. Med J Aust [Internet]. 2007 Oct [cited 2022 May 26];187(S7). Available from: https://onlinelibrary.wiley.com/doi/abs/10.5694/j.1326-5377.2007.tb01335.x

10. Kelleher I, Cannon M. Psychotic-like experiences in the general population: characterizing a high-risk group for psychosis. Psychol Med. 2011 Jan;41(1):1–6.

11. Alvaro PK, Roberts RM, Harris JK. A Systematic Review Assessing Bidirectionality between Sleep Disturbances, Anxiety, and Depression. Sleep. 2013 Jul 1;36(7):1059–68.

12. Kelleher I, Keeley H, Corcoran P, Lynch F, Fitzpatrick C, Devlin N, et al. Clinicopathological significance of psychotic experiences in non-psychotic young people: evidence from four population-based studies. Br J Psychiatry. 2012 Jul;201(1):26–32.

13. Hauser M, Correll CU. The Significance of At-Risk or Prodromal Symptoms for Bipolar I Disorder in Children and Adolescents. Can J Psychiatry. 2013 Jan;58(1):22–31.

14. Faedda GL, Marangoni C, Serra G, Salvatore P, Sani G, Vázquez GH, et al. Precursors of Bipolar Disorders: A Systematic Literature Review of Prospective Studies. J Clin Psychiatry. 2015 May 27;76(05):614–24.

15. Addington AM, Gallo JJ, Ford DE, Eaton WW. Epidemiology of unexplained fatigue and major depression in the community: The Baltimore ECA Follow-up, 1981–1994. Psychol Med. 2001 Aug;31(6):1037–44.

16. Suter P, Kuipers J, Moffa G, Beerenwinkel N. Bayesian Structure Learning and Sampling of Bayesian Networks with the R Package BiDAG. J Stat Softw. 2023 Jan 28;105:1–31.

17. Gelman A, Carlin JB, Stern HS, Dunson DB, Vehtari A, Rubin DB. Bayesian Data Analysis. Third edition. 2013.

18. Højsgaard S. Graphical Independence Networks with the gRain Package for R. J Stat Softw. 2012 Feb 28;46:1–26.

19. Revelle W. psych: Procedures for Psychological, Psychometric, and Personality Research [Internet]. 2023. Available from: https://cran.r-project.org/web/packages/psych/citation.html

20. Kim S. ppcor: Partial and Semi-Partial (Part) Correlation [Internet]. 2015. Available from: https://cran.r-project.org/web/packages/ppcor/index.html

21. Borsboom D, Deserno MK, Rhemtulla M, Epskamp S, Fried EI, McNally RJ, et al. Network analysis of multivariate data in psychological science. Nat Rev Methods Primer. 2021 Aug 19;1(1):1–18.

22. Oisín R, Bringmann LF, Schuurman NK. The Challenge of Generating Causal Hypotheses Using Network Models. Struct Equ Model Multidiscip J. 2022 Nov 2;29(6):953–70.
